# A case of *ALK*-rearranged non–small cell lung cancer that responded to ceritinib after development of resistance to alectinib

**DOI:** 10.18632/oncotarget.25143

**Published:** 2018-05-01

**Authors:** Yosuke Makuuchi, Hidetoshi Hayashi, Koji Haratani, Junko Tanizaki, Kaoru Tanaka, Masayuki Takeda, Kazuko Sakai, Shigeki Shimizu, Akihiko Ito, Kazuto Nishio, Kazuhiko Nakagawa

**Affiliations:** ^1^ Department of Medical Oncology, Kindai University Faculty of Medicine, Osaka-Sayama, Osaka 589-8511, Japan; ^2^ Department of Hematology, Graduate School of Medicine, Osaka City University, Abeno, Osaka 545-8585, Japan; ^3^ Department of Genome Biology, Kindai University Faculty of Medicine, Osaka-Sayama, Osaka 589-8511, Japan; ^4^ Department of Pathology, Kindai University Faculty of Medicine, Osaka-Sayama, Osaka 589-8511, Japan

**Keywords:** non–small cell lung cancer (NSCLC), L1196M, anaplastic lymphoma kinase (ALK) fusion gene, ceritinib, alectinib

## Abstract

The second-generation anaplastic lymphoma kinase (ALK) tyrosine kinase inhibitors (TKIs) alectinib and ceritinib are standard treatment options for patients with non–small cell lung cancer (NSCLC) positive for *ALK* fusion genes. However, almost all patients eventually develop resistance to these drugs. We here report a case of *ALK*-rearranged NSCLC that developed resistance to alectinib but remained sensitive to ceritinib. The L1196M mutation within the *ALK* fusion gene was detected after failure of consecutive treatment with crizotinib and alectinib, but no other mechanism underlying acquired resistance to ALK-TKIs was found to be operative. Given the increasing application of ALK-TKIs to the treatment of patients with *ALK*-rearranged NSCLC, further clinical evaluation is warranted to provide a better understanding of the mechanisms of acquired resistance to these agents and to inform treatment strategies for such tumors harboring secondary mutations.

## INTRODUCTION

Rearrangements of the anaplastic lymphoma kinase (ALK) gene have been detected in 3% to 7% of non–small cell lung cancers (NSCLCs) [1, 2]. Such tumors are oncogene addicted and are therefore usually sensitive to treatment with ALK tyrosine kinase inhibitors (TKIs). Alectinib (CH5424802) and ceritinib (LDK378) are highly selective second-generation ALK-TKIs that have recently been developed to overcome acquired resistance to the first-generation ALK-TKIs crizotinib. Alectinib shows pronounced anticancer activity against *ALK* fusion–positive NSCLC cells that harbor the most common crizotinib resistance mutations [3, 4]. It also reduced the risk of disease progression or death by 66% compared with crizotinib in a randomized phase III trial for ALK-TKI–naïve patients with *ALK*-rearranged NSCLC [5, 6]. Despite its marked efficacy, however, almost all patients treated with alectinib eventually develop resistance to this drug, with no therapeutic strategy having been shown to be effective for overcoming such resistance. Ceritinib has also manifested robust efficacy in crizotinib-resistant patients, being associated with a median progression-free survival (PFS) of 5.4 months in a randomized phase III trial [7]. In addition, it has shown an efficacy similar to that of alectinib in ALK-TKI–naïve patients with *ALK*-rearranged NSCLC, with a median PFS of 16.6 months [8]. However, the efficacy of ceritinib for patients previously treated with alectinib has remained unknown. We here report a case of ALK-rearranged NSCLC that progressed after treatment with crizotinib and alectinib possibly due to L1196M secondary mutation, and subsequently manifested tumor shrinkage during treatment with ceritinib.

## CASE PRESENTATION

**Figure 1:**
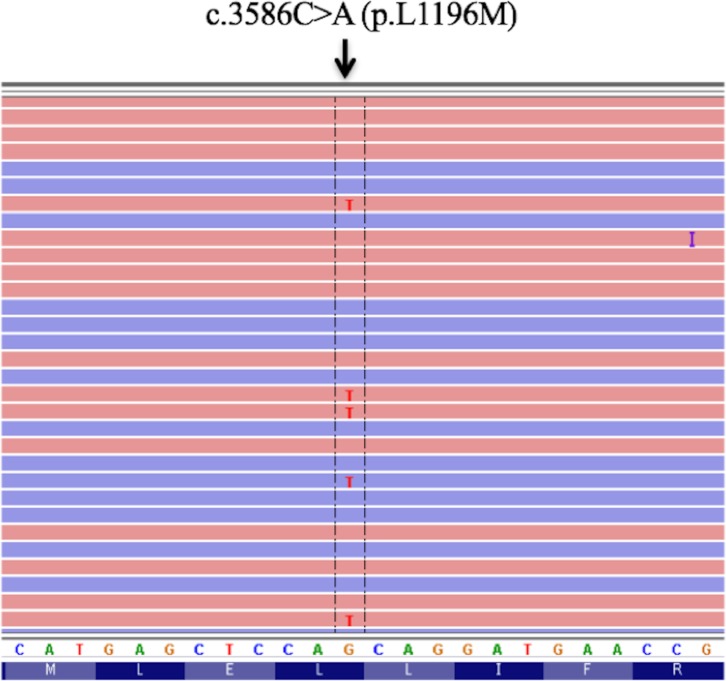
Sequence trace for cDNA encoding the L1196M mutation of the ALK kinase domain that was derived from the NSCLC of the patient

**Figure 2:**
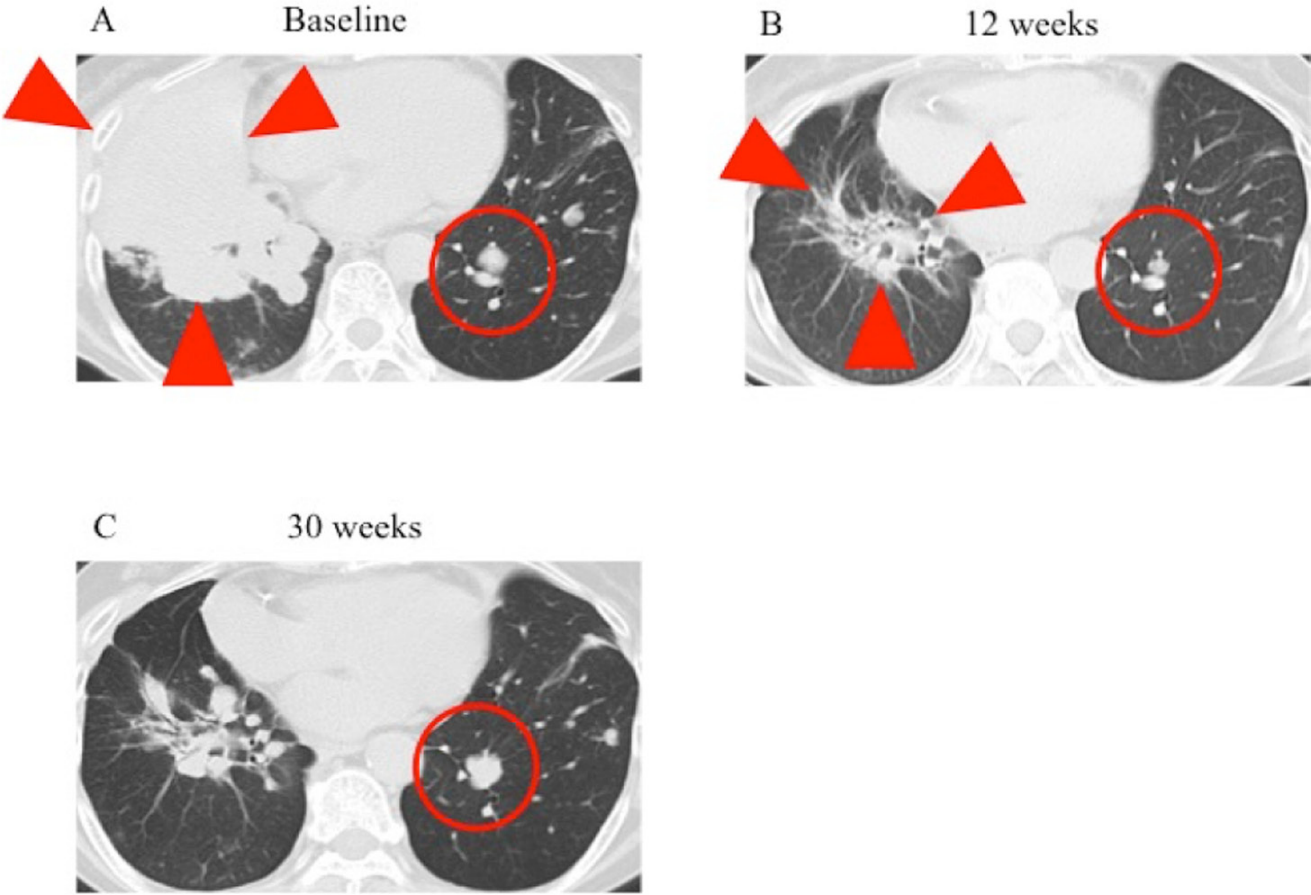
CT findings for the lung lesions of the patient during ceritinib treatment CT scans of the lung lesions (red arrowheads and circles) were performed immediately before (**A**) as well as 12 (**B**) and 30 (**C**) weeks after the onset of ceritinib treatment.

A 58-year-old woman who was a light smoker was diagnosed with stage IIA adenocarcinoma of the left lung. The tumor was removed by surgical resection and was found to harbor an *ALK* fusion gene without L1196M mutation by genomic analysis. Three years after surgery, the patient experienced disease recurrence as lung metastases. She was treated with 3 cycles of bevacizumab plus carboplatin and paclitaxel as a first-line drug treatment, following 22 cycles of bevacizumab and pemetrexed as the maintenance therapy, but 2 years later computed tomography (CT) again revealed progression of her pulmonary disease. Crizotinib was then administered 200 mg twice daily and induced marked tumor regression with manageable toxicity for 19 months. Subsequent progression of lung metastases was followed by treatment with alectinib at 300 mg twice daily, which yielded a partial response. Despite the emergence of multiple pulmonary metastases as revealed by CT at 8 months after the onset of alectinib treatment, the patient continued alectinib therapy for another 6 months beyond disease progression, given the manageable toxicity profile and slowly progression of disease. A bronchoscopic biopsy was performed to identify the genomic change underlying the acquired resistance of the lung metastases to alectinib. Targeted sequencing of DNA encoding the entire ALK kinase domain with the Ion AmpliSeq Colon and Lung Cancer Research Panel v2 (Life Technologies–Thermo Fisher Scientific, Waltham, MA, USA) revealed the L1196M mutation (Figure 1). Immunohistochemistry analysis showed no staining of phosphorylated Insulin-like growth-factor-1 receptor (IGF-1R) which is recognized as the resistance factor about alectinib. Alectinib was discontinued, and chemotherapy was commenced using nivolumab (3 mg/kg, 3-weekly for 4 cycles) followed by 60 mg/m^2^ of docetaxel for 1 cycle. Since there was no alternative treatment with tolerable toxicity, she was rechallenged with alectinib at 300 mg twice daily for 2 months until disease progression. Five months after discontinuation of the initial alectinib treatment, CT again revealed progression of lung metastases (Figure 2A). Ceritinib was then administered at 750 mg once daily. Although the patient experienced nausea and diarrhea that required a dose reduction to 450 mg once daily, she was well managed with ceritinib and CT at 12 weeks after treatment onset revealed tumor regression (Figure 2B). After 30 weeks of ceritinib administration, however, CT showed that the lung metastases had again progressed (Figure 2C) and brain magnetic resonance imaging revealed brain metastasis.

## DISCUSSION

We here describe a case of *ALK*-rearranged NSCLC that underwent disease progression during alectinib treatment, was found to harbor the L1196M mutation of ALK, and then showed a response to ceritinib treatment. Genomic analysis of the primary lung tumor both at diagnosis and after progression of alectinib revealed that the L1196M change was an acquired mutation with crizotinib or alectinib. *ALK*-rearranged NSCLC is initially sensitive to ALK-TKIs but eventually develops resistance to these drugs through various mechanisms. In general, these mechanisms fall into two classes: (1) genetic alterations of the *ALK* fusion gene itself including secondary mutation and gene amplification, both of which limit the ability of the drugs to inhibit kinase activity; and (2) changes to alternative signaling pathways—such as those mediated by the epidermal growth factor receptor, c-KIT, or IGF-1R—that allow ALK-related signaling to be bypassed [9, 10]. In the present case, we performed target sequencing and pathological evaluation with immunohistochemistry. We did not detect amplification of the *ALK* fusion gene, any bypass track, or other secondary mutations such as I1171 or V1180L that are known to be associated with sensitivity to ceritinib and resistance to alectinib [11, 12] (data not shown), suggesting that, whereas the L1196M mutation within the *ALK* fusion gene confers resistance to alectinib, it might not affect sensitivity to ceritinib.

The most common secondary mutation in the kinase domain of *ALK* fusion genes is the gatekeeper substitution L1196M [13]. Our report implied that the L1196M mutation in *ALK*-rearranged NSCLC is related to disease progression during alectinib treatment and ceritinib can overcome this resistance in the clinical setting. Clinical studies suggested that both of alectinib and ceritinib were effective in patients with *ALK*-rearranged NSCLC harboring L1196M mutation [14, 15]. More recently, in phase 2 Japanese clinical trial of ceritinib for alectinib resistance NSCLC, treatment of ceritinib achieved the tumor shrinkage for the patient harboring L1196M mutation [16]. *ALK*-rearranged NSCLC cell lines harboring various types of secondary *ALK* mutations were recently assayed for sensitivity to ALK-TKIs *in vitro* [17]. In the case of cells expressing an *ALK* fusion gene harboring the L1196M mutation, both crizotinib and alectinib showed lower potency in inhibiting ALK phosphorylation (median inhibitory concentrations of 339 and 117.6 nM, respectively) than ceritinib (9.3 nM). These results demonstrated that *ALK* fusion gene harboring the L1196M mutation conferred higher level of resistance to crizotinib and alectinib than ceritinib. These preclinical results are thus consistent with the present case, which responded to ceritinib after failure of alectinib treatment and was found by a next-generation sequencing assay to harbor the L1196M mutation as the mechanism of resistance.

In summary, we report a case of *ALK*-rearranged NSCLC that developed resistance to alectinib but remained sensitive to ceritinib. This case suggests that the L1196M mutation contributed to the differential response to alectinib versus ceritinib. As far as we are aware, our report is the first to show that the L1196M mutation in *ALK*-rearranged NSCLC is related to disease progression during alectinib treatment and that ceritinib can overcome this resistance in the clinical setting. Further studies are thus warranted to provide a better understanding of this phenomenon and to inform treatment strategies for *ALK*-rearranged NSCLC positive for secondary mutations after progression during alectinib treatment.

## Abbreviations

ALK: anaplastic lymphoma kinase
TKI: tyrosine kinase inhibitor
NSCLC: non–small cell lung cancer
PFS: progression-free survival
CT: computed tomography
IGF-1R: insulin-like growth-factor-1 receptor
